# Elevated Serum Lipopolysaccharides and Intrinsic Factor Autoantibodies Correlate With Macrocytic Anemia Among People With HIV

**DOI:** 10.1002/iid3.70378

**Published:** 2026-02-22

**Authors:** Daniel Amakye, Samuel Hammond, Osbourne Quaye, Upal Roy, Peter Puplampu, Vincent Ganu, Emmanuel Ayitey Tagoe

**Affiliations:** ^1^ Department of Biochemistry, Cell & Molecular Biology/West African Centre for Cell Biology of Infectious Pathogens (WACCBIP) University of Ghana Legon Accra Ghana; ^2^ Department of Clinical Chemistry Accra Technical University Ridge Accra Ghana; ^3^ Department of Health and Biomedical Science The University of Texas Rio Grande Valley Brownsville Texas USA; ^4^ Department of Medicine, University of Ghana Medical School University of Ghana Korle Bu Accra Ghana; ^5^ Department of Medical Laboratory Sciences, School of Biomedical and Allied Health Sciences University of Ghana Korle Bu Accra Ghana

**Keywords:** HIV, intrinsic factor autoantibodies, macrocytic anemia, serum lipopolysaccharide, translocation

## Abstract

**Introduction:**

Human immunodeficiency virus (HIV) infection is associated with various comorbidities, including macrocytic anemia, though the role of the infection is unclear. HIV has been implicated in microbial translocation and altered immune responses. This study aimed to establish the relationship between gut microbial translocation and immune response, and macrocytic anemia among people with HIV (PWH).

**Materials and Methods:**

Fifty‐five PWH on combination antiretroviral therapy (cART) were age‐matched with 55 HIV‐negative individuals. Demographic data and blood samples were collected from February to July 2023. Hematological indices, including red blood cell (RBC) count, hemoglobin concentration, and mean corpuscular volume (MCV), were measured. Serum lipopolysaccharides (LPS) and intrinsic factor autoantibodies (IFAA) were measured using ELISA.

**Results:**

The prevalence of macrocytic anemia was significantly higher in the HIV cohort (43.6%) compared to HIV‐negative individuals (1.8%), (*p* < 0.0001). PWH exhibited higher microbial translocation, characterized by elevated LPS levels (*p* < 0.0001). PWH with macrocytic anemia showed significantly increased serum LPS and IFAA levels compared to those without anemia (*p* < 0.001). Intrinsic factor autoantibodies correlated positively with systolic (*r* = 0.49, *p* < 0.001) and diastolic blood pressures (*r* = 0.31, *p* < 0.01), as well as LPS (*r* = 0.60, *p* < 0.001) and MCV (*r* = 0.65, *p* < 0.001).

**Conclusion:**

This study reports for the first time elevated lipopolysaccharides and intrinsic factor autoantibodies among PWH, and the lipopolysaccharides and intrinsic factor autoantibodies strongly correlated with mean corpuscular volume in the patients. Targeting serum lipopolysaccharides and intrinsic factor autoantibodies may offer a novel therapeutic strategy.

## 1 Introduction

The human immunodeficiency virus (HIV) remains a global public health threat, infecting 39.9 million people across the globe in 2023 [1]. The advent of combination antiretroviral therapy (cART) has been effective in reducing HIV‐associated mortality, transforming the infection from a terminal illness into a chronic, manageable type [2]. Despite the success in suppressing viral loads and improving immune function, the infection and treatment regimen have been associated with comorbidities. Co‐morbidities in people with HIV (PWH) are well characterized and may increase mortality in the individuals if not managed. PWH with weak antioxidant proteins were found to show increased hypertension and other risk factors of cardiovascular diseases [3, 4]. The well‐reported comorbidity among PWH is cardiovascular disease, which has been attributed to cART [5].

The hematological derangement is another established non‐AIDS‐related comorbidity among PWH. The anemia in HIV‐infected individuals is multifactorial, which includes nutritional deficiency, drug‐induced bone marrow suppression and many other common factors [6]. Chronic HIV infection triggers a state of persistent immune activation, where pro‐inflammatory cytokines like interleukin‐6 (IL‐6) induce hepcidin production, leading to “anemia of chronic disease” by sequestering iron and inhibiting erythropoiesis [7]. In this regard, HIV infection‐related immune dysregulation is one of the key factors that directly contributes to anemia [8]. The prevalence of anemia varies among HIV populations, with higher rates among infants, women, and those in low‐ and middle‐income countries (LMIC) [9]. Anemia is an important prognostic marker to assess the progression of the infection as it complicates the infection and reduces the bioavailability of drugs [10].

Several types of anemia have been reported in PWH; however, little is known about the prevalence of pernicious anemia in PWH [11]. The condition arises primarily from the destruction of gastric parietal cells, which produce an intrinsic factor, a glycoprotein essential for vitamin B12 absorption in the ileum [12]. Vitamin B12 deficiency poses significant health risks, particularly for PWH, who are already vulnerable to anemia and other comorbidities [13]. The deficiency can manifest in various forms, from subclinical reductions in serum levels to overt pernicious anemia, which impairs oxygen delivery. The anemia characterized by larger red blood cells is referred to as macrocytic anemia [14]. High mean corpuscular volume (MCV) is a surrogate marker for macrocytic anemia, and HIV patients, even on cART, were found to show elevated MCV [15].

HIV exerts deleterious effects on multiple systems, including the immune and digestive systems [16]. A common manifestation of HIV infection‐associated damage of the gastrointestinal tract (GIT) is leaky gut syndrome, marked by an increased intestinal permeability [17]. The GIT, particularly the gut‐associated lymphoid tissue (GALT), is a primary site of HIV replication, and the rapid replication compromises the structural and functional integrity of the gut epithelium [18]. Damage to the gut epithelium drives the translocation of gut microbes or microbial products, such as lipopolysaccharides (LPS), into systemic circulation [19]. Host response to the circulating microbial products results in immune system dysregulation and activation of chronic inflammation, known to promote adverse health outcomes and heightened susceptibility to comorbidities [20]. Microbial translocation is known to drive cardiometabolic conditions and can be due to host‐dysregulated physiological responses [21]. Increased levels of translocated microbial products are linked with inflammatory response and autoimmune disorders [22]. Autoimmune disorders, including immune thrombocytopenic purpura, inflammatory myositis (IM), sarcoidosis, Guillain‐Barré syndrome (GBS), myasthenia gravis and Graves' disease have been reported in PWH [23]. However, altered autoantibody production against intrinsic factor and macrocytic anemia among PWH is not well documented. In addition, the relationship between circulatory bacterial LPS largely released through possible leaky gut is not well investigated in the context of HIV infection. This current study aims to establish a relationship between serum LPS, intrinsic factor autoantibodies and macrocytic anemia in PWH.

## 2 Methods

### 2.1 Study Design and Participants

A case‐control study was conducted at the Fevers Unit of the Korle‐Bu Teaching Hospital (KBTH), a premier and largest referral hospital in Ghana. The Fevers Unit, under the Department of Medicine and Therapeutics, is responsible for registering and managing all individuals diagnosed with HIV. Healthy individuals were recruited as controls from the hospital environment. A total of 55 PWH on cART were age‐matched with 55 HIV seronegative individuals in this study. Demographic data and blood samples were collected from February to July 2023, and hematological analysis was done immediately after the blood sampling. Pregnant and breastfeeding mothers, patients with other chronic conditions including hepatitis and cancers, and patients with stomach ulcers and nutritional deficiencies were excluded from the study. Similarly, study participants on broad‐spectrum antibiotics were not included in the study. All the study participants consented to be part of the study by signing an informed consent. Committee on Human Research, Publication and Ethics, College of Health Sciences, Kwame Nkrumah University of Science and Technology (CHRPE AP/007/23) approved this study.

### 2.2 Data Collection and Blood Sample Processing

Whole blood sample was collected from each participant and divided into EDTA and gel separator tubes. The samples in the EDTA tubes were mixed thoroughly and transported for hematological analysis. Socio‐demographic data as well as medical history were collected using a well‐structured questionnaire. The samples in the separator tubes were processed for serum and stored at −80°C until ready to be used. Clinical records were reviewed to obtain data on current medication regimens, including cART and routine nutritional supplementation. All participants had been on ART for a minimum of 6 months prior to enrollment, and the majority were taking multivitamins to support nutritional status.

### 2.3 Laboratory Analysis

A fully automated hematology analyzer, (BC 760 Mindray Hematology Analyzer, China) was used to analyze the hematological parameters, hemoglobin (HB), erythrocyte count, white blood cell count, platelet count, hematocrit, mean platelet volume, MCV, mean cell hemoglobin, mean cell hemoglobin concentration, and total and differential white blood cell count following manufacturer's protocol.

Serum LPS concentration in HIV seropositive patients and seronegative controls was measured by a competitive enzyme immunoassay technique using an ELISA kit (MyBioSource Inc., San Diego, CA, USA) following the manufacturer's protocol. A competitive enzyme immunoassay technique using Guinea pig Anti Intrinsic Factor Autoantibody (AIFA) ELISA kit (MyBioSource Inc., San Diego, CA, USA) following the manufacturer's protocol was used to measure serum intrinsic factor autoantibody concentrations. All assays were read on the Varioskan LUX multimode microplate reader (Thermo Scientific, USA) operating on the SkanIt software at an absorbance of 450 nm. All samples were measured in duplicates, and the mean concentration was calculated for data analysis. The laboratory analyses were carried out at the Virology and Cancer Laboratory, Department of Biochemistry, Cell and Molecular Biology/West African Centre for Cell Biology of Infectious Pathogens (WACCBIP), University of Ghana, Accra, Ghana.

### 2.4 Statistical Analysis

Data were entered into an Excel spreadsheet and exported to IBM SPSS Statistics 20 for analysis. Continuous data were summarized as mean and standard deviation, and the Student t‐test was used to determine the significance level of the mean difference. Qualitative data were presented as percentages. Chi‐square ( *χ* ^2^) was used for comparison of proportions and odds ratio (OR) for association. One‐way ANOVA was used to compare the means of more than two groups, and a correlation matrix was used to determine the strength of the relationship between variables. All statistical tests were analyzed at a 95% confidence level. *p* < 0.05 was considered statistically significant.

## 3 Results

### 3.1 Characterization of Cohort Participants

The socio‐demographic and clinical parameters of the study participants are presented in Table 1. Marital status distribution among the study groups was different (*p* < 0.001) while sex and educational status were evenly distributed (*p* > 0.05). The prevalence of high MCV was higher in the patients (43.6%) than in the HIV seronegative individuals (1.8%). Table 2 shows the clinical and hematological parameters. Both systolic and diastolic blood pressures were significantly elevated, although body mass index was reduced in PWH compared to the HIV seronegative individuals (*p* < 0.01). The mean infection duration was 16.70 ± 12.61 months.

**Table 1:** Socio‐demographic and RBCs status of the study participants.

Parameters	PWH (*N* = 55)	HIV seronegative (*N* = 55)	Chi‐square (*χ* ^2^)	*p* value
Sex*: n* (%)				
Female	26 (47.0)	23 (42.0)	0.3312	0.5649
Male	29 (53.0)	32 (58.0)		
Marital status: *n* (%)				
Single	19 (34.5)	31 (56.4)	14.94	< 0.001*
Married	19 (34.5)	22 (40.0)		
Divorced/widowed	17 (31.0)	2 (3.6)		
Education level *n* (%)				
Basic/none	18 (32.7)	11 (20.0)	2.373	0.3054
Secondary	25 (45.5)	31 (56.4)		
Tertiary	12 (21.8)	13 (23.6)		
Macrocytosis	24 (43.6)	1 (1.8)		< 0.0001*

*Note:* N = population size, n = size of subgroup. Categorical data is expressed as frequency and percentage, n (%). Macrocytosis, MCV > 100 fL. The Fisher exact test was used to compare proportions less than 5.

* *p*‐value < 0.05 was considered statistically significant.

**Table 2:** Clinical and hematological parameters of the study participants.

Parameters	PWH (*N* = 55)	HIV seronegative (*N* = 55)	95% CI of mean diff.	*p* value
Age (years)	46.42 ± 15.32	45.45 ± 11.46	−4.07−5.99	0.7095
Infection duration (mths)	16.70 ± 12.61	—	—	—
BMI (kg/m^2^)	21.67 ± 1.71	23.23 ± 3.47	−2.62−(−0.50)	< 0.01*
DBP (mmHg)	86.18 ± 9.88	76.45 ± 16.14	5.27−14.19	< 0.001*
SBP (mmHg)	141.22 ± 13.03	126.29 ± 17.38	9.05−20.80	< 0.001*
HB (g/dL)				
Males	11.16 ± 2.09	13.68 ± 2.44	1.55−3.84	< 0.001*
Females	10.34 + 2.34	11.20 ± 2.32	−0.21−2.00	0.1113
RBC				
Males	4.22 ± 0.78	4.81 ± 0.64	0.20−0.98	< 0.01*
Females	4.02 ± 0.70	4.50 ± 0.59	0.15−0.80	< 0.01*
MCV (fL)	99.51 ± 10.29	85.39 ± 6.67	−17.54−(−10.71)	< 0.0001*

*Note:* N = population size. Data is presented as mean ± standard deviation and Student's *t*‐test used for comparison. SBP= Systolic blood pressure, DBP = Diastolic blood pressure, BMI= Body mass index. (%). Anemia was defined as hemoglobin (HB) value < 13 g/dL for adult males and < 12 g/dL for non‐pregnant women. Mean corpuscular volume (MCV). Microcytosis was defined as MCV < 80 fL and macrocytosis, MCV > 100 fL.

* *p*‐value < 0.05 was considered statistically significant.

### 3.2 Antiretroviral Regimen of Participants

**Figure 1:**
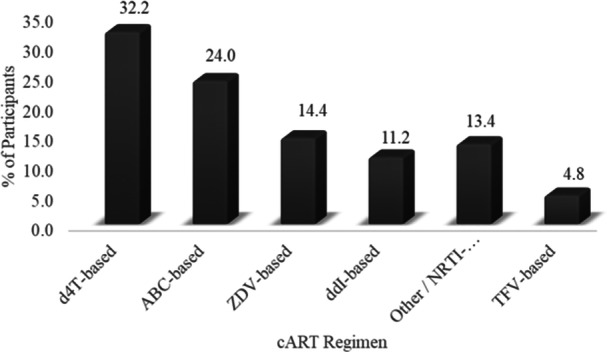
Distribution of antiretroviral regimen among participants. Participants were receiving a heterogeneous range of antiretroviral therapy regimens. The most common combination was Stavudine+efavirenz+darunavir. Stavudine, followed by Abacavir are the most common nucleoside reverse transcriptase inhibitors used in the therapy.

Participants were receiving a heterogeneous range of antiretroviral therapy regimens at the time of recruitment, as presented in Figure 1.

### 3.3 Comparison of Microbial Translocation and Autoantibody Levels

**Figure 2:**
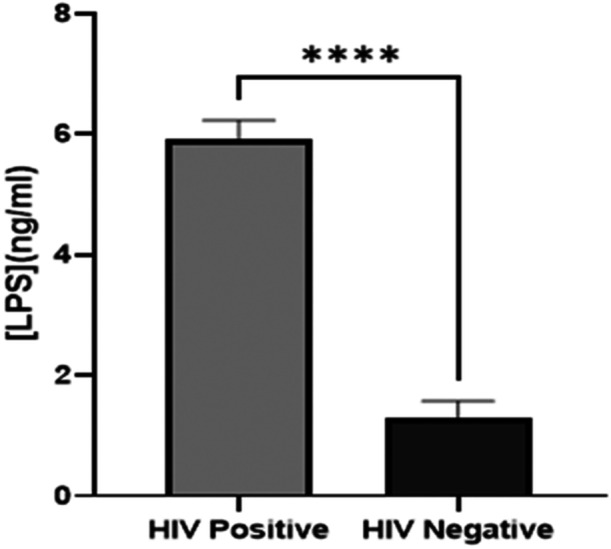
Comparison of serum levels LPS between PWH and non‐infected participants. ****p < 0.0001. *p* < 0.05 was considered statistically significant.

**Figure 3:**
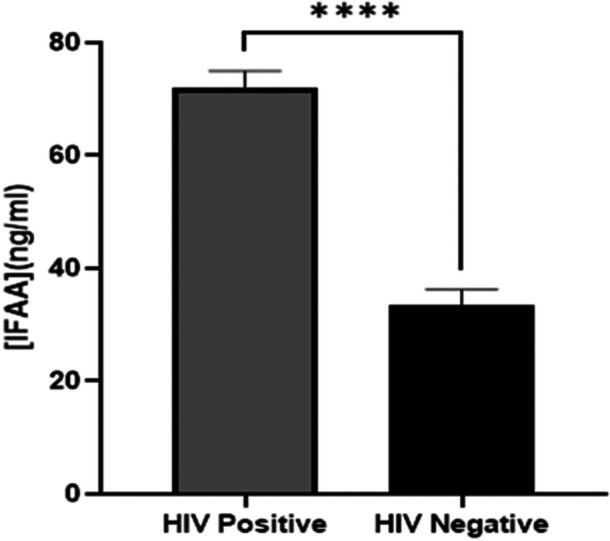
Comparison of serum intrinsic factor autoantibody levels between PLWH and non‐infected participants. ****p < 0.0001. *p* < 0.05 was considered statistically significant.

Serum concentrations of LPS and intrinsic factor autoantibodies (IFAA) among HIV‐infected patients and non‐infected participants are presented in Figures 2 and 3. PWH had significantly higher concentrations of the LPS and antibodies than their non‐infected counterpart (*p* < 0.0001).

### 3.4 Comparison of Serum LPS and Intrinsic Factor Autoantibodies Among PWH With High MCV

**Figure 4:**
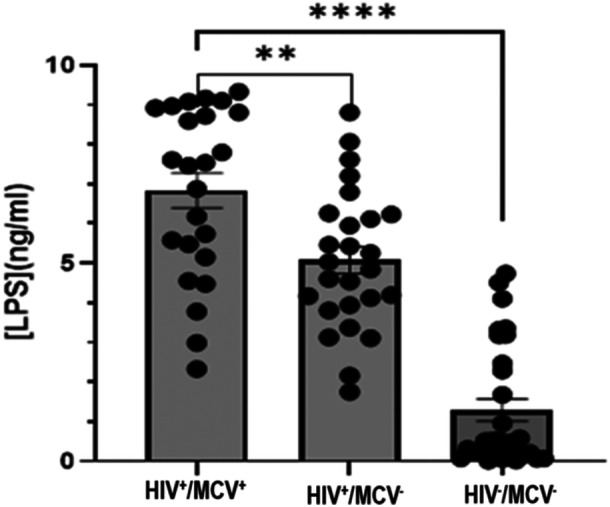
Serum concentrations of LPS in the subgroups. HIV^+^/MCV^+^; HIV seropositive with macrocytic anemia, HIV^+^/MCV^‐^; HIV seropositive without macrocytic anemia, and HIV^‐^/MCV^‐^; HIV seronegative without macrocytic anemia. **p < 0.01, ****p < 0.0001. *p* < 0.05 was considered statistically significant.

**Figure 5:**
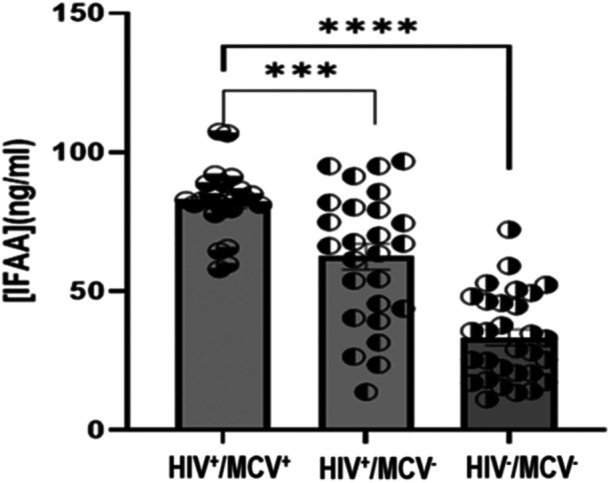
Serum concentrations of intrinsic factor autoantibodies in the subgroups. HIV^+^/MCV^+^; HIV seropositive with macrocytic anemia, HIV^+^/MCV^‐^; HIV seropositive without macrocytic anemia, and HIV^‐^/MCV^‐^; HIV seronegative without macrocytic anemia. ***p < 0.001, ****p < 0.0001. *p* < 0.05 was considered statistically significant.

Comparison of serum levels of LPS and intrinsic factor autoantibodies among PWH with high MCV are shown in Figures 4 and 5, respectively. The circulating levels of LPS and intrinsic factor autoantibodies (IFAA) in the HIV cohort with high MCV were significantly higher than those infected with HIV but without macrocytic anemia (*p* < 0.001) and the HIV seronegative group (*p* < 0.0001). In addition, the HIV seropositive group without macrocytic anemia had statistically significantly higher LPS and IFAA than the seronegative group (*p* < 0.001).

### 3.5 LPS and Intrinsic Factor Autoantibodies Correlate With Clinical and Hematological Parameters

Correlation of LPS and intrinsic factor autoantibodies with clinical and hematological parameters is presented in Table 3. Serum LPS positively and strongly correlated with systolic (*r* = 0.48, *p* < 0.001) and diastolic (*r* = 031, *p* < 0.001) blood pressures, and MCV (*r* = 0.72, *p* < 0.01). However, LPS negatively and significantly correlated with RBC (−0.31, *p* < 0.01) and Hb (*r* = −0.31, *p* < 0.01) levels. Levels of LPS strongly and positively correlated with IFAA (0.60, *p* < 0.0001). Serum IFAA correlated positively and significantly with SBP (r = 0.49, *p* < 0.001) and DBP (*r* = 0.31, *p* < 0.001). Nevertheless, IFAA showed a reverse relation with RBC (*r* = −0.14, *p* > 0.05) and HB (*r* = −0.24, *p* < 0.05). IFAA level positively and significantly correlated with MCV (*r* = 0.65, *p* < 0.0001).

**Table 3:** Correlation matrix of serum concentration of lipopolysaccharide, intrinsic factor autoantibodies and clinical parameters.

Variable/Pearson correlation		Age	BMI	SBP	DBP	RBC	HB	MCV	LPS	IFAA
Age	*r*	1.000	0.095	0.229	0.091	−0.005	0.037	0.146	0.028	0.099
	*p*		ns	0.0414*	ns	ns	ns	ns	Ns	ns
BMI	*r*	0.095	1.000	0.042	0.073	0.110	0.013	−0.032	0.032	−0.167
	*p*	ns		ns	ns	ns	ns	ns	Ns	ns
SBP	*r*	0.229	0.042	1.000	0.661	−0.073	0.058	0.471	0.542	0.488
	*p*	0.0414*	ns		< 0.0001*	ns	ns	< 0.0001*	< 0.0001*	< 0.0001*
DBP	*r*	0.091	0.073	0.661	1.000	0.083	0.083	0.209	0.360	0.306
	*p*	ns	ns	< 0.0001*		ns	ns	ns	< 0.0010*	0.0057*
RBC	*r*	−0.005	0.110	−0.073	0.083	1.000	0.242	−0.272	−0.311	−0.137
	*p*	ns	ns	ns	ns		0.0307*	0.0147*	0.0049**	ns
HB	*r*	0.037	0.013	0.058	0.083	0.242	1.000	−0.235	−0.311	−0.240
	*p*	ns	ns	ns	ns	0.0307*		0.0358*	0.0045*	0.0320*
MCV	*r*	0.146	−0.032	0.471	0.209	−0.272	−0.235	1.000	0.724	0.652
	*p*	ns	ns	< 0.0001*	ns	0.0147*	0.0358*		< 0.0001*	< 0.0001*
[LPS]	*r*	0.028	0.032	0.542	0.360	−0.311	−0.314	0.724	1.000	0.601
	*p*	ns	ns	< 0.0001*	< 0.001*	0.0049*	0.0045*	< 0.0001*		< 0.0001*
[IFAA]	*r*	0.099	−0.167	0.488	0.306	−0.137	−0.240	0.652	0.601	1.000
	*p*	ns	ns	< 0.0001*	0.0057*	ns	0.0320*	< 0.0001*	< 0.0001*	

Abbreviations: (BMI), Body mass index; (DBP), Diastolic blood pressure; (HB), Hemoglobin; (IFAA), Intrinsic factor autoantibodies; (LPS), Lipopolysaccharides; (MCV), Mean corpuscular volume; (r), Pearson correlation coefficient; (RBC), Red blood cell counts; (SBP), Systolic blood pressure.

* *p* < 0.05 was considered statistically significant.

## 4 Discussion

The current study reports an altered hematological profile, increased translocation of gastric contents and altered immunological response in PWH. The results of this study identified a reduced red blood cell count and hemoglobin concentration in PWH. Additionally, patients showed markedly increased MCV, a surrogate marker for macrocytic anemia, compared with the HIV seronegative group.

An analysis of blood parameters in PWH revealed reduced RBC counts compared to healthy controls [24]. A study that assessed hematological parameters as predictors of morbidity in PWH reported a decrease in RBC count and hemoglobin concentration with decreasing immunological status [25]. However, an improvement in hematological parameters and a decrease in RBC count were reported in HIV patients 3 months after dolutegravir‐based cART [26]. A study in Sao Paolo, Brazil, reported a similar improvement in hematological parameters with cART [27]. A decrease in RBC counts and hemoglobin levels has been proposed to arise from impaired erythropoiesis, the virus's direct effect on progenitor cells in the bone marrow and cART drugs like Zidovudine [28]. The study participants were receiving a wide range of antiretroviral therapy regimens, including zidovudine‐containing and non–zidovudine combinations, with no single regimen predominating. Although certain antiretroviral drugs are known to induce macrocytosis, the heterogeneous ART exposure observed suggests that treatment‐related effects alone are unlikely to fully account for the hematological abnormality. Macrocytic cells are characteristic of abnormal RBC production and anemia, usually as a result of the absence of vitamin B12, folate, and some medications [29].

Elevated levels of serum LPS were found in PWH compared with HIV seronegative controls, and the level was higher in PWH with macrocytic anemia compared with PWH without macrocytic anemia. A study that analyzed leaky gut‐related products in the circulation of PWH reported increased serum concentration of LPS [30]. There is a complex association between LPS level in the blood and macrocytic anemia that has not been investigated before. The present study, for the first time, has shown that there is a strong and positive correlation between these two factors in PWH treated with cART. A longitudinal study that assessed LPS as a marker for translocation reported a time‐dependent increase in the levels of the endotoxin [31]. Furthermore, increased plasma levels of LPS were correlated with insulin resistance in HIV patients [32]. Nevertheless, a study reported the normalization of serum LPS within 2 years of effective cART [33]. A leaky gut in HIV patients leads to translocation of microbial and microbial products such as LPS from the gut into circulation [34].

Generally, there were elevated intrinsic factor autoantibodies in the circulation of HIV patients on cART, and the level was higher among patients with macrocytic anemia than those without macrocytic anemia. An *in vitro* study reported anti‐CNS antibodies in HIV patients with neurological complications [35]. Furthermore, there are case reports of anti‐N‐methyl‐d‐aspartate Receptor antibodies resulting in encephalitis and autoimmune hepatitis [36]. Intrinsic factor produced by the parietal cells in the gut facilitates the absorption of vitamin B12 [12]. The intrinsic factor autoantibodies also suggest an autoimmune‐mediated mechanism surrounding the macrocytic anemia reported in this study. It is important to note that the high prevalence of macrocytic anemia (43.6%) observed here occurred despite the use of multivitamins containing B‐complex. While routine supplementation is intended to prevent nutritional deficiency, the presence of elevated IFAA suggests a “functional” rather than a purely nutritional deficit. IFAA specifically interferes with the formation of the Vitamin B12‐intrinsic factor complex, thereby impairing absorption in the ileum regardless of oral intake. This suggests that in PWH, systemic inflammation and the resulting autoimmune response may render standard oral supplementation insufficient for maintaining normal erythropoiesis. There are, however, no reports of intrinsic factor autoantibodies in people living with HIV in low‐ and middle‐income countries. The elevated autoantibody levels observed in this study were associated with increased markers of microbial translocation, suggesting a potential link between gut barrier dysfunction and immune dysregulation involving intrinsic factor autoantibodies

In addition to the above‐mentioned relationship, the current study also observed that serum intrinsic factor autoantibodies in PWH correlated positively and strongly with LPS, and both factors strongly and positively correlated with MCV in the patients.

Circulatory LPS is known to interact with TLR on immune cells, producing inflammatory cytokines and increasing costimulatory molecules on antigen‐presenting cells [37]. In this context, elevated LPS levels have been widely linked to chronic immune activation and inflammatory states observed in people living with HIV, which are implicated in the development of non‐AIDS‐related complications. Elevated LPS is also known to cause a chronic systemic inflammation, which disrupts erythropoiesis [38]. Furthermore, the interaction of LPS with toll‐like receptors has also been known to release cytokines, which can interfere with DNA synthesis in developing erythrocytes, and the production of larger and less matured RBCs cells [39].

The strong positive correlation found between LPS and IFAA in this study suggests that chronic microbial translocation may serve as the primary trigger for the immune dysregulation that leads to the production of these autoantibodies.

Despite these findings, this study has limitations that warrant caution in interpretation. As a cross‐sectional pilot study with a relatively small sample size, we establish significant correlations but cannot definitively confirm biological causality. Furthermore, direct serum measurements of Vitamin B12 and folate were not performed. While the presence of IFAA and the context of routine multivitamin use strongly point toward a functional deficiency, future longitudinal studies incorporating direct B12 levels and markers like methylmalonic acid (MMA) are needed. Additionally, while we excluded patients with known liver disease, a more detailed assessment of dietary habits and alcohol consumption in larger cohorts would further strengthen the generalizability of these results. Also, although antiretroviral regimens were documented, the study was not powered to perform stratified analyses based on individual drug combinations, including zidovudine‐containing therapies.

## 5 Conclusion

The current study reports for the first time elevated LPS and intrinsic factor autoantibodies with high MCV in PWH on cART. The LPS and intrinsic factor autoantibodies strongly correlated with MCV in the patients. This suggests that serum LPS and intrinsic factors autoantibodies could be surrogate biomarkers for macrocytic anemia in PWH. Further investigations with a larger sample size to explore the possibilities for autoantibodies neutralization in a specialized therapy are warranted.

## Author Contributions

Emmanuel Ayitey Tagoe, Osbourne Quaye: Conception and design; Daniel Amakye, Vincent Ganu and Samuel Hammond: Acquisition of data; Emmanuel Ayitey Tagoe, Osbourne Quaye and Peter Puplampu: Data analysis and interpretation of data; Upal Roy, Samuel Hammond and Daniel Amakye: Drafted the manuscript; All authors reviewed and approved the final manuscript.

## Conflicts of Interest

The authors declare no conflicts of interest.

## Data Availability

The authors confirm that the data supporting the findings of this study are available within the article.
